# Repeated vapor ethanol exposure induces transient histone modifications in the brain that are modified by genotype and brain region

**DOI:** 10.3389/fnmol.2015.00039

**Published:** 2015-08-05

**Authors:** Andrey Finegersh, Carolyn Ferguson, Seth Maxwell, David Mazariegos, Daniel Farrell, Gregg E. Homanics

**Affiliations:** Departments of Anesthesiology, Pharmacology and Chemical Biology, University of PittsburghPittsburgh, PA, USA

**Keywords:** alcoholism, histone modification, histone modifying enzymes, ethanol, GABA-A receptor

## Abstract

**Background:** Emerging research implicates ethanol (EtOH)-induced epigenetic modifications in regulating gene expression and EtOH consumption. However, consensus on specific epigenetic modifications induced by EtOH has not yet emerged, making it challenging to identify mechanisms and develop targeted treatments. We hypothesized that chronic intermittent EtOH (CIE) induces persistent changes in histone modifications across the cerebral cortex (CCx), nucleus accumbens (NAc), and prefrontal cortex (PFC), and that these histone modifications are altered in a knock-in mouse strain with altered sensitivity to EtOH.

**Methods:** C57BL/6J (B6) mice and α1SHLA knockin mice on a B6 background were exposed to 16 h of vapor EtOH or room air followed by 8 h of room air for 4 consecutive days and sacrificed at multiple time points up to 72 h following exposure. Histone modifications were assessed using Western blot and dot blot. RT-qPCR was used to study expression of chromatin modifying enzymes in NAc and PFC.

**Results:** In NAc, CIE significantly increased acetylation of histone subunit H3 at lysine 9 (H3K9ac) but not lysine 14 (H3K14ac) or lysine 27 (H3K27ac). In PFC, CIE significantly increased H3K9ac but not H3K14 or H3K27ac. There were no significant changes at 8 or 72 h after EtOH exposure in either NAc or PFC. CIE was also associated with increased expression of *Kat2b, Kat5*, and *Tet1* in NAc but not PFC. In CCx, CIE had a significant effect on levels of H3K18ac; there was also a significant effect of the α1SHLA mutation on levels of H3K27me3, H3K14ac, and H3K18ac as well as a trend for H3S10pK14ac.

**Conclusions:** The EtOH-induced histone modifications observed were transient and varied significantly between brain regions. A genetic mutation that altered sensitivity to EtOH was associated with altered induction of histone modifications during CIE. These results have implications for studying EtOH-induced histone modifications and EtOH sensitivity.

## Introduction

Repeated ethanol (EtOH) exposure is associated with molecular adaptations in the brain that underlie its potential for dependence (Robinson and Berridge, 1993; Koob, 2003). Notably, EtOH exposure leads to widespread changes in gene expression across several brain structures in both rodents (Kerns et al., 2005; Wolen et al., 2012) and humans (Mayfield et al., 2002; Ponomarev et al., 2012), with the number of altered genes dependent on the brain region studied (Melendez et al., 2012). Despite a considerable number of studies demonstrating EtOH-induced changes in gene expression in the brain, mechanisms by which EtOH selectively regulates these genes are only recently emerging.

Gene expression is regulated by epigenetic mechanisms, which refer to a broad group of modifications to chromatin that do not alter nucleotide sequence and include DNA methylation, modifications to histone N-terminal tails, and noncoding RNAs (Kouzarides, 2007). In particular, modifications to histone N-terminal tails regulate the accessibility of DNA to transcription factors by altering the affinity between DNA and histones. Among the best characterized modifications, acetylation of lysine residues on histone N-terminal tails is associated with gene activation while methylation of these same residues is associated with gene repression. Additionally, these histone modifications are catalyzed by a large group of histone modifying enzymes and are rapidly inducible and reversible, forming a critical component of cellular adaptation to its environment (Smith and Shilatifard, 2010). Because of their plasticity and role in regulating gene expression in response to an environmental stressor, histone modifications have been increasingly studied for their role in drug-induced behavior and addiction (Robison and Nestler, 2011).

Emerging evidence indicates EtOH exposure and withdrawal lead to global changes in histone modifications across several brain structures that are critical for EtOH-related behaviors and drinking. In the amygdala, EtOH exposure is associated with increased histone acetylation and withdrawal is associated with decreased histone acetylation (Pandey et al., 2008; Sakharkar et al., 2012; Moonat et al., 2013); moreover, modulating histone deacetylase (HDAC) activity in the amygdala reduces anxiety-like behaviors during withdrawal and alters EtOH consumption (Moonat et al., 2013; You et al., 2014). Potentiation of the mesolimbic pathway by EtOH is also associated with altered histone modifications in the ventral tegmental area (VTA) (Arora et al., 2013) and nucleus accumbens (NAc) (Simon-O'Brien et al., 2014; Sprow et al., 2014). In the hippocampus, EtOH-induced histone modifications regulate expression of brain derived neurotrophic factor (BDNF) exons (Stragier et al., 2014).

Globally modulating histone modifying also alters behavior. Pretreatment with an HDAC inhibitor decreased aversion and increased extinction time on a conditioned place aversion assay involving EtOH as well as potentiating the effect of EtOH on histone acetylation in the prefrontal cortex (PFC) (Pascual et al., 2012). While studies have focused on increased histone acetylation following EtOH exposure, acute EtOH is associated with both histone acetylation and deacetylation at gene promoters in the cerebral cortex (CCx), suggesting induction of multiple chromatin remodeling pathways (Finegersh and Homanics, 2014).

While a diverse group of studies on epigenetic mechanisms of EtOH has emerged, several critical questions remain to be addressed. Notably, published studies of EtOH-induced histone modifications have focused on acute and chronic EtOH exposure as well as withdrawal. However, alcohol use disorder (AUD) in humans is associated with chronically relapsing patterns of EtOH drinking (for review, see Koob, 2013), so that persistence of epigenetic modifications following a binge episode may be important for reinforcing further consumption. In mice, chronic intermittent EtOH (CIE) is also associated with escalation of EtOH drinking up to 1 week following the last dose of EtOH (Lopez and Becker, 2005; Griffin et al., 2009). Published studies have also generally focused on a single critical brain region or network for studying EtOH-induced epigenetic effects; however, brain regions have distinct transcriptional profiles in response to EtOH exposure (Kerns et al., 2005; Melendez et al., 2012) and even two sub-structures of the amygdala have distinct induction of histone acetylation following EtOH exposure (Pandey et al., 2008). Genetic diversity also contributes to altered susceptibility to AUD in humans. Similarly, mouse strains show a wide range of voluntary EtOH consumption (Belknap et al., 1993) and EtOH-induced behaviors (Rustay et al., 2003). These behavioral differences are related to distinct patterns of EtOH-induced gene expression in strains with high vs. low EtOH preference (Kerns et al., 2005), suggesting differences in EtOH-induced epigenetic modifications.

This paper addresses whether EtOH-induced changes to histone modifications persist beyond acute EtOH withdrawal, whether altered histone modifications generalize across brain structures, and if genotype affects EtOH-induced histone modifications. To study these factors, we examined induction of histone acetylation in NAc, and PFC following CIE through 72 h of withdrawal in wild type mice. In a separate experiment, we studied EtOH-induced histone modifications in knock-in mice containing two point mutations in the α1 subunit of the GABA_A_ receptor (S270H, L277A) (α1SHLA). This mutation reduces sensitivity of post-synaptic GABA_A_ receptors to EtOH (Borghese et al., 2006; Werner et al., 2006), decreases sensitivity to motor-impairing effects of EtOH (Werner et al., 2006), increases sensitivity to the anxiolytic effects of EtOH (Werner et al., 2006), and significantly alters gene expression in CCx compared to WT littermates (Harris et al., 2011). This experiment will also investigate basal and EtOH-induced histone modifications in this genetically engineered rodent strain. Overall, results from these experiments are expected to elucidate new epigenetic effects and improve design of mechanistic studies of EtOH exposure.

## Methods

All experiments were approved by the Institutional Animal Care and Use Committee of the University of Pittsburgh and conducted in accordance with the National Institutes of Health Guidelines for the Care and Use of Laboratory Animals. Mice were habituated to the University of Pittsburgh animal facility for at least 1 week prior to initiation of experiments. Mice were housed under 12 h light/dark cycles and had *ad libitum* access to food and water.

### Experiment 1

This experiment was performed using 8- to 12-week-old, EtOH-naïve, specific pathogen free male C57BL/6J mice from the Jackson Laboratory (20–30 g). Male mice were used because the estrous cycle influences EtOH drinking behaviors in females (Roberts et al., 1998; Ford et al., 2002). Mice were randomly divided into groups receiving either chronic intermittent vapor EtOH (E) or room air (C). For exposures, mice were injected with either 1 g/kg EtOH (0.01 ml/g of 10% EtOH in 0.9% saline i.p.) or an equivalent volume of 0.9% saline i.p. Immediately following injection, mice were placed in one of two identical custom-built vapor chambers (16" × 16" × 24" constructed from 0.5" plexiglass). One chamber was used to deliver room air and the other vaporized EtOH. Flow rate, vaporization temperature, and exposure time were optimized to achieve consistent blood EtOH concentrations (BEC) without the use of pyrazole. Room air was flowed into two heated Erlenmeyer flasks at a rate of 8 L/min; one flask received EtOH at a rate of 160 μl/min by a syringe pump (Harvard apparatus, Holliston, MA, USA) while the other flask received no EtOH. Mice were placed in vapor chambers immediately after injections overnight from 17:00 to 09:00 followed by room air from 09:00 to 17:00 for 4 consecutive days. Temperature of the chambers was monitored daily and averaged 78°F at the end of 16 h of exposure. Blood was collected from the tail vein immediately following the first EtOH exposure. BEC in plasma were measured using an Analox EtOH analyzer (AM1, Analox Instruments, London, UK).

Mice were sacrificed immediately following the fourth night of vapor EtOH (chronic EtOH), 8 h later (8 h withdrawal), or 72 h later (72 h withdrawal). NAc and PFC were extracted immediately using an ice cold adult mouse brain slicer matrix with 1 mm coronal section slice intervals (Zivic Instruments, Pittsburgh, PA, USA). PFC was extracted on the first slice where the cortex was visible. NAc was defined as tissue wrapped around the anterior commissure inferior to the caudate and superior to the olfactory tubercle. Tissue was flash frozen and stored at −80°C until processing.

For Western blots, histone proteins were extracted using the Epigentek Total Histone Extraction Kit according to the manufacturer's protocol (Epigentek, Farmingdale, NY, USA). Protein concentration was quantified using a Bradford assay and 3 μg of protein were loaded into a 12% Novex Tris-glycine gel (Life Technologies, Carlsbad, CA, USA). Proteins were transferred to a nitrocellulose membrane using the iBlot system (Life Technologies). For antibody blots, membranes were blocked by Odyssey buffer (Licor Biosciences, Lincoln, NE, USA) for 1 h at room temperature. Then membranes were incubated with primary antibody overnight at 4°C. Antibodies were obtained from the Acetyl-Histone H3 Antibody Sampler Kit (Cell Signal Technologies, #9927) and included H3K9ac (#9649), H3K14ac (#7627), H3K27ac (#8173), and Total H3 (#4499). Blots were washed 3 × in TBST for 5 min each then an anti-rabbit fluorescent secondary antibody (Licor Biosciences) was added and incubated for 1 h at room temperature. The secondary antibody was washed 3 × in TBST for 5 min each and fluorescence was visualized using the Odyssey Infrared Imaging System (Licor Biosciences). Membranes were stripped three times using stripping buffer (LiCor Biosciences) between incubations with primary antibody and visualized afterward to ensure there was no detectable antibody signal between blots.

Acetyl-histone bands were normalized to the total intensity of histone subunit H3. All samples were run in duplicate and the average normalized intensity between duplicates used for analysis. A One-Way ANOVA with Bonferroni *post-hoc* tests was used to compare histone modifications between groups.

For RT-qPCR experiments, total RNA was isolated using TRIzol (Life Technologies) according to the manufacturer's protocol, purified with on-column DNase digestion using the RNeasy Mini kit (Qiagen, Venlo, Netherlands), and 1 μg of RNA was synthesized into cDNA using reverse transcriptase (RT) (Bio-Rad, Hercules, CA, USA). For quantification, samples were run with each primer pair and SYBR green fluorescent master mix (Bio-Rad) and visualized using a Bio-Rad iCycler. All primers were optimized for 90–110% efficiency at the following conditions: 10 min at 95°C (initial denaturation) followed by 40 cycles of 30 s at 95°C (denaturation), 1 min at 60°C (annealing), and 30 s at 72°C (extension). Primer sequences for β-actin, *Hdac2, Hdac11, Kat2b, Kat5, Ehmt2*, and *Tet1* are shown in Table 1. Threshold cycle (Ct) values were calculated for each well and duplicate values averaged. The difference between specific genes and β-actin (ΔCt) was calculated for each animal and normalized to the average of saline-treated animals (ΔΔCt). Fold change over room air controls was calculated for each animal using the following formula: 2^−ΔΔCt^. A Two-Way repeated measures ANOVA with Bonferroni *post-hoc* tests was used to compare gene expression between groups.

**Table 1:** **Primer sequences for RT-qPCR experiments**.

**Gene**	**Forward primer (5^′^ → 3^′^)**	**Reverse primer (5^′^ → 3^′^)**
β*-actin*	TCATGAAGTGTGACGTTGACATCCGT	CCTAGAAGCATTTGCGGTGCACGATG
*Hdac2*	GACATATGAGACTGCAGTTGC	ACCTCCTTCACCTTCATCCTC
*Hdac11*	AATGGGGCAAGGTGATCAAC	AGCCACCACCAACATTGATG
*Kat2b*	TACCTCTTCACCTGCGTCCACAAA	TCACACCCTGTTCAATACTGGGCT
*Kat5*	AGGACATCAGTGGCCGAAAG	GTGATCTGGACCGGGATTGG
*Ehmt2*	TGCCTATGTGGTCAGCTCAG	GGTTCTTGCAGCTTCTCCAG
*Tet1*	CGAAAGAACAGCCACCAGAT	TTGCTCTTCTTCCCCATGAC

### Experiment 2

α1SHLA knockin mice and littermate controls were on a mixed C57Bl/6J × Strain 129SvJ background that had been backcrossed to C57BL/6J for three generations. Mice were randomly divided into groups receiving either vapor EtOH (E) or room air (C). For exposures, mice were given the alcohol dehydrogenase inhibitor pyrazole (68 mg/kg i.p.) followed by a priming dose of 1.5 g/kg EtOH (0.02 ml/g of 7.5% EtOH in 0.9% saline i.p.) or an equivalent volume of saline i.p. and then placed into the vapor chambers for 16 h of EtOH vapor exposure (16:00–08:00) followed by 8 h in room air (08:00–16:00). Pyrazole is an alcohol dehydrogenase inhibitor that was used to maintain high BECs during ethanol vapor exposure. Compressed air at rate of 8 L/min was flowed over two heated flasks; one flask received EtOH at a rate of 110 μl/min by a syringe pump (Harvard apparatus) while the other flask received no EtOH; the vapor was then passed into two identical custom-built vapor chambers (16" × 16" × 24" constructed from 0.5" plexiglass). The exposure process was repeated for a total of four cycles.

For each treatment group, mice were sacrificed and brain tissue collected at three endpoints: 1 h into the first exposure (acute exposure), immediately following the fourth exposure (chronic exposure), and 9 h into the withdrawal period after the fourth exposure (withdrawal). At sacrifice, CCx was quickly dissected, flash-frozen in liquid nitrogen, and stored at −80°C. For the EtOH treatment group, six animals per endpoint were designated for each genotype (WT, KI), for a total of 36 samples. For the room air group, two animals per endpoint were designated within genotype; the three endpoints (six animals) were pooled into one room air group as the control for each genotype, for a total of 12 samples.

Histone proteins were extracted using the Epigentek Total Histone Extraction Kit according to the manufacturer's protocol (Epigentek) and concentration of the lysate was determined by a Bradford assay. Relative changes in modified H3 levels were measured using the dot blot assay. In this assay, 4–8 μg of histone protein lysate per sample was spotted in random order on nitrocellulose in a 6 × 8 grid either by hand or using a vacuum manifold. Membranes were blocked in Odyssey buffer (Licor Biosciences) and incubated overnight simultaneously with primary total- and modified-H3 antibodies. Membranes were then incubated with fluorescent secondary antibodies and visualized in two channels (680 and 800 nm) using the Odyssey Infrared Imaging System (Licor Biosciences). Modified histone levels were determined using the integrated signal intensities in the following manner. First, a ratio of the modified H3 to total H3 intensity was calculated for each sample. This ratio was normalized to the average of the room air group, within genotype. The average of the six normalized ratios was then calculated for each endpoint. Three replicate blots were done for each modified histone and a Two-Way ANOVA with Bonferroni *post-hoc* tests was performed on the averaged replicates to assess the effect of endpoint and genotype.

## Results

### Experiment 1

**Figure 1:**
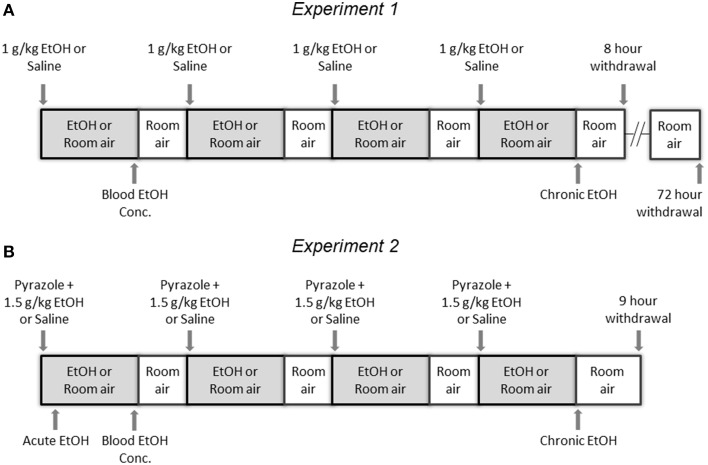
**Experimental Design**. **(A)** In Experiment 1, mice were injected with either 1 g/kg EtOH or saline then immediately placed into a vapor EtOH chamber or room air for 16 h overnight (1700 to 0900) for 4 consecutive days; they were sacrificed immediately following the final vapor EtOH exposure (chronic EtOH), 8 h following the final vapor EtOH exposure (8 h withdrawal), or 72 h following the final vapor EtOH exposure and their NAc and PFC were flash frozen and stored for analysis. **(B)** In Experiment 2, mice were injected with pyrazole and either 1.5 g/kg EtOH or saline then immediately placed into a vapor EtOH chamber or room air for 16 h overnight (1700 to 0900) for 4 consecutive days; they were sacrificed 1 h into the first day (acute EtOH), immediately following the final vapor EtOH exposure (chronic EtOH), or 9 h following the final vapor EtOH exposure (9 h withdrawal) and their CCx was flash frozen and stored for analysis. In both experiments, blood EtOH concentrations were collected by tail nick immediately following the first vapor EtOH session.

The goal of this experiment was to compare levels of histone acetylation following CIE and withdrawal in NAc and PFC. Mice were injected with either 1 g/kg EtOH or saline then immediately placed into a vapor EtOH chamber or room air for 16 h overnight for 4 consecutive days; they were sacrificed immediately following the final vapor EtOH exposure (chronic EtOH), 8 h afterward (8 h withdrawal), or 72 h afterward (Figure 1A). BECs were measured after the first exposure cycle and averaged 238 ± 19.2 mg/dl (mean ± SEM). NAc and PFC were removed and used to compare levels of histone modifications and gene expression of chromatin modifying enzymes to room air controls. Western blot statistical comparisons are summarized in Tables 2, 3.

**Table 2:** **Summary of ANOVAs of histone modifications by brain region**.

**Brain Region**	**H3K9ac**	**H3K14ac**	**H3K18ac**	**H3K27ac**	**H3S10phK14ac**	**H3K27me3**
Nucleus Accumbens	EtOH^**^	EtOH^**^	–	EtOH^**^	–	–
Prefrontal Cortex	EtOH^**^	N.S.	–	N.S.	–	–
Cerebral Cortex	N.S.	G^**^	G + EtOH^**^	–	G^*^	G^***^

EtOH, effect of EtOH treatment; G, effect of genotype; N.S., not significant—not done,

* p < 0.1,

** p < 0.05,

*** *p < 0.01*.

**Table 3:** **Summary of Bonferroni ***post-hoc*** tests for Experiment 1**.

	**Nucleus accumbens**			**Prefrontal cortex**		
**Treatment**	**H3K9ac**	**H3K14ac**	**H3K27ac**	**H3K9ac**	**H3K14ac**	**H3K27ac**
Chronic EtOH	0.03	0.06	0.14	0.02	0.12	0.39
8 h withdrawal	0.99	0.13	0.11	0.92	0.58	0.51
72 h withdrawal	0.99	0.86	0.99	0.99	0.99	0.99

**Figure 2:**
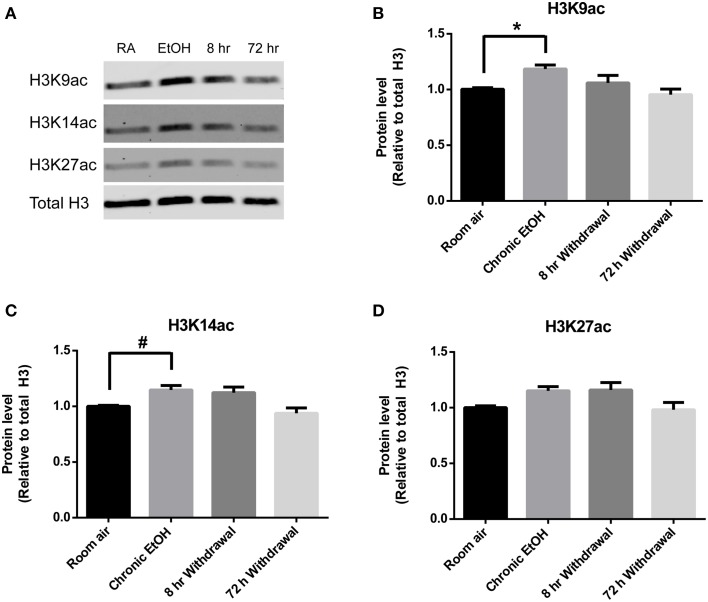
**Chronic intermittent EtOH transiently potentiates histone acetylation in NAc. (A)** Western blot showing one sample from 6/group analyzed in duplicate. Western blot revealed **(B)** a significant effect of EtOH exposure on H3K9ac (*p* < 0.05) and increased levels immediately following EtOH exposure, **(C)** a significant effect of EtOH on levels of H3K14ac (*p* < 0.01) and a trend for increased levels immediately following EtOH exposure, and **(D)** a significant effect of EtOH on levels of H3K27ac (*p* < 0.05). ^*^p < 0.01, #*p* = 0.056, *n* = 6/group, data presented as mean ± SEM.

Levels of H3K9ac, H3K14ac, and H3K27ac were assessed in NAc using Western blot (Figure 2A). There was a significant effect of EtOH exposure on levels of H3K9ac (*F* = 4.55, *p* < 0.05); *post-hoc* analysis revealed increased levels of H3K9ac following chronic EtOH (*p* < 0.05) (Figure 2B). There was also a significant effect of EtOH exposure on level levels of H3K14ac (*F* = 6.61, *p* < 0.01); *post-hoc* analysis revealed a trend for an increase in H3K14ac following chronic EtOH (*p* = 0.056) (Figure 2C). There was also a significant effect of EtOH exposure on levels of H3K27ac (*F* = 3.56, *p* < 0.05) but no significant findings on *post-hoc* analysis (Figure 2D).

**Figure 3:**
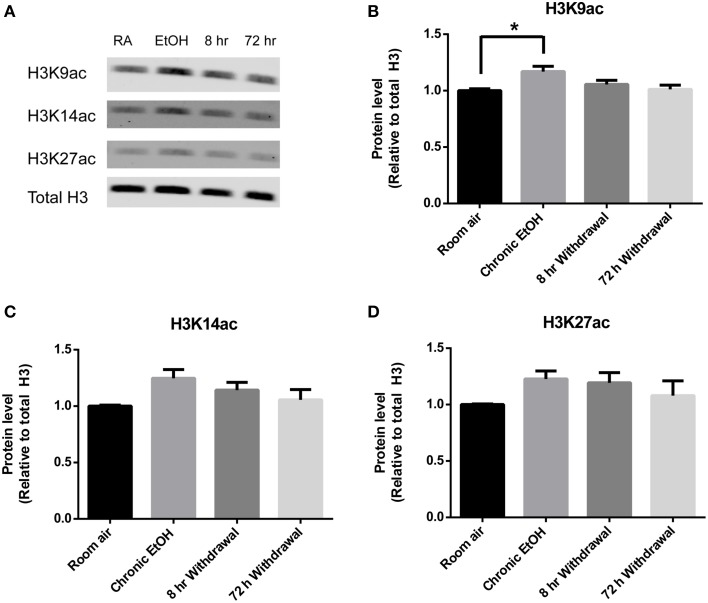
**Chronic intermittent EtOH transiently potentiates histone acetylation in the PFC. (A)** Western blot showing one sample from 5/group analyzed in duplicate. Western blot revealed **(B)** a significant effect of EtOH exposure on H3K9ac (*p* < 0.05) and increased levels immediately following EtOH exposure but no significant effect of EtOH exposure on **(C)** H3K14ac or **(D)** H3K27ac levels in mPFC. ^*^p < 0.01, *n* = 5/group, data presented as mean ± SEM.

Histone acetylation was also assessed by Western blot in PFC (Figure 3A). There was a significant effect of EtOH exposure on levels of H3K9ac (*F* = 4.028, *p* < 0.05); *post-hoc* analysis revealed increased levels of H3K9ac following chronic EtOH (*p* < 0.05) (Figure 3B). There were no significant effects of EtOH exposure on levels of H3K14ac (Figure 3C) or H3K27ac (Figure 3D) in the mPFC.

**Figure 4:**
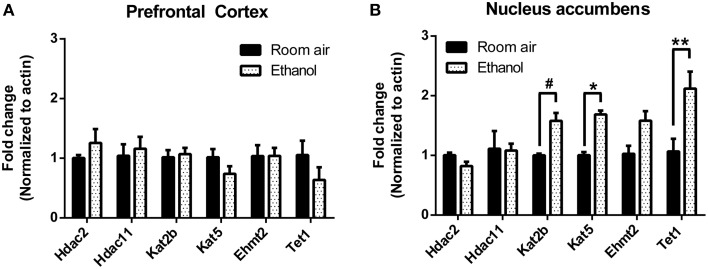
**Chronic intermittent EtOH alters expression of select chromatin modifying enzymes in NAc but not PFC**. Gene expression of a diverse set of chromatin modifying enzyme expression was assessed using RT-qPCR. **(A)** There was no significant effect of EtOH on expression of enzymes in the mPFC. **(B)** There was a significant effect of chronic EtOH exposure on gene expression (*p* < 0.05) and *post-hoc* analysis revealed significantly increased expression of *Kat5* and *Tet1* as well as a trend for increased expression of *Kat2b*. ^**^p < 0.01, ^*^p < 0.05, #*p* = 0.08, *n* = 4–5/group, data presented as mean ± SEM.

Chromatin modifications are carried out by a diverse group of enzymes that are rapidly induced by environmental stimuli (Smith and Shilatifard, 2010). We performed RT-qPCR in the chronic EtOH group to assess expression of chromatin modifying enzymes that may be involved in mediating the epigenetic effects of EtOH. These included the histone deacetylases (HDAC) *Hdac2* and *Hdac11* previously shown to be down-regulated by acute EtOH (Finegersh and Homanics, 2014), the histone acetyltransferases *Kat2b* and *Kat5* that are associated with the cAMP-response element binding protein (CREB), the histone lysine methyltransferase *Ehmt2*, and a component of an active DNA demethylation pathway *Tet1*. In PFC, there was no significant effect of chronic EtOH exposure on expression of these enzymes (Figure 4A). In NAc, there was a significant effect of chronic EtOH exposure on gene expression [*F*_(1, 7)_ = 11.66, *p* < 0.05]; *post-hoc* analysis revealed significantly increased expression of *Kat5* (*p* < 0.05) and *Tet1* (*p* < 0.01) and a trend for increased expression of *Kat2b* (*p* = 0.08) (Figure 4B).

### Experiment 2

Based on results from Experiment 1, we tested whether observed changes in histone modifications in NAc and PFC generalized to CCx and were affected by gene mutations conferring changes in EtOH-induced gene expression and behavior. To study these effects, we compared WT and α1SHLA knockin littermates. Mice were injected with pyrazole and either 1.5 g/kg EtOH or saline then placed into a vapor EtOH chamber or room air chamber for 16 h overnight for 4 consecutive days; they were sacrificed 1 h into the first day (acute EtOH), immediately following the final vapor EtOH exposure (chronic EtOH), or 9 h afterward (9 h withdrawal) (Figure 1B). BECs were measured after the first exposure cycle and were in the range of 150–300 mg/dl. We chose histone modifications to study based on those previously reported to be involved in addiction. Notably, our previous study showed that acute EtOH alters H3K27me3 and H3K9,14ac at specific gene promoters in CCx (Finegersh and Homanics, 2014). H3S10pK14ac was studied because it is induced in the striatum following cocaine administration (Stipanovich et al., 2008) and in hepatocytes following EtOH exposure (Lee and Shukla, 2007) but there are no published studies that describe its levels in CCx following EtOH exposure. H3K18ac was studied because it is enriched at the transcriptional start site of actively transcribed gene promoters (Wang et al., 2008; Ernst et al., 2011). Dot blot statistical results are summarized in Tables 2, 4.

**Table 4:** **Summary of Bonferroni ***post-hoc*** tests for Experiment 2**.

**Treatment**	**H3K9ac**	**H3K14ac**	**H3K18ac**	**H3S10 phK14ac**	**H3K27me3**
**WILD TYPE**					
Acute EtOH	0.99	0.99	0.99	0.99	0.99
Chronic EtOH	0.99	0.99	0.02	0.99	0.99
9 h withdrawal	0.99	0.99	0.99	0.99	0.99
**α1 SHLA**					
Acute EtOH	0.99	0.54	0.99	0.52	0.06
Chronic EtOH	0.99	0.65	0.61	0.99	0.86
9 h withdrawal	0.99	0.61	0.72	0.28	0.99

**Figure 5:**
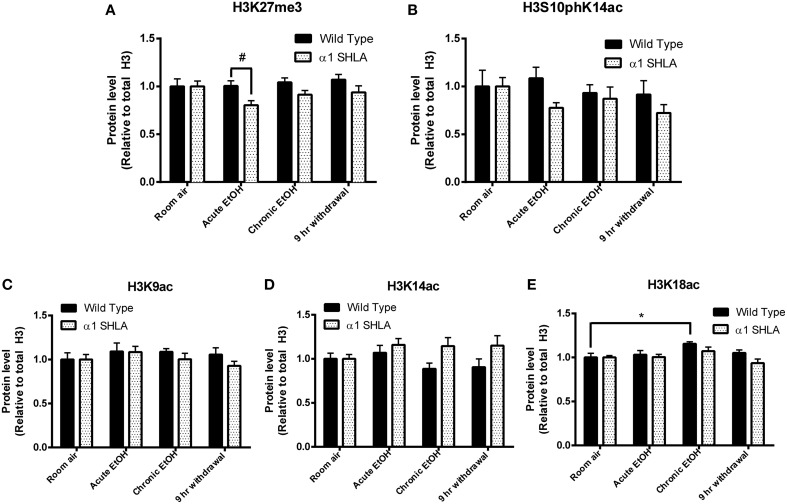
**Altered histone modifications in CCx of α1SHLA mice following EtOH exposure**. Levels of histone modifications in CCx were measured using dot blot and revealed **(A)** a significant effect for genotype (*p* < 0.01) for H3K27me3 and trend for decreased levels in α1 SHLA mice compared to WT mice following acute EtOH, **(B)** a trend for an effect of genotype on levels of H3S10pK14ac (*p* = 0.09), **(C)** no effect on levels of H3K9ac, **(D)** an effect of genotype on levels of H3K14ac (*p* < 0.05), and **(E)** an effect of both genotype (*p* < 0.05) and EtOH exposure (*p* < 0.05) on levels of H3K18ac and a significant increase in H3K18ac following CIE in WT mice. ^*^p < 0.05, #*p* = 0.07, *n* = 6/group, data presented as mean ± SEM.

Using dot blot, we identified a significant effect of genotype on levels of H3K27me3 [*F*_(1, 40)_ = 8.12, *p* < 0.01] but no effect of EtOH exposure or interaction; *post-hoc* analysis revealed a nonsignificant trend for decreased levels of H3K27me3 after acute EtOH exposure in α1 SHLA compared to WT littermates (*p* = 0.07) (Figure 5A). There was also a nonsignificant trend for an effect of genotype on levels of H3S10pK14ac [*F*_(1, 40)_ = 3.02, *p* = 0.09] but no effect of EtOH exposure, interaction, or significant *post-hoc* effects (Figure 5B).

We also assessed levels of histone acetylation in CCx by studying H3K9ac, H3K14ac, and H3K18ac using dot blot. There was no significant effect of EtOH treatment or genotype on levels of H3K9ac (Figure 5C). There was a significant effect of genotype on levels of H3K14ac [*F*_(1, 40)_ = 6.57, *p* < 0.05] but no effect of EtOH exposure, interaction, or significant *post-hoc* effects (Figure 5D). We also identified a significant effect for both genotype [*F*_(1, 40)_ = 4.28, *p* < 0.05] and EtOH exposure [*F*_(3, 40)_ = 4.13, *p* < 0.05] on levels of H3K18ac but no significant interaction; *post-hoc* analysis revealed increased levels of H3K18ac in the chronic EtOH group compared to room air exposure in WT mice (*p* < 0.05) (Figure 5E).

## Discussion

Our results demonstrate that for the histone modifications studied, changes induced by EtOH are transient, differ significantly between brain regions, and are altered in knockin mice that have altered responses to EtOH. Importantly, Experiment 2 of this study identified a significant effect of the GABA_A_ receptor α1SHLA mutation on CCx levels of H3K27me3, H3K14ac, and H3K18ac as well as a trend for H3S10pK14ac, suggesting epigenetic modifications underlie observed differences in gene expression (Harris et al., 2011) and basal behavior (Werner et al., 2006) in this strain. Additionally, Experiment 1 of this study identified a large effect of chronic EtOH on histone acetylation in NAc but not in PFC, reflecting sensitivity of different brain regions to EtOH-induced histone modifications.

It is notable that at least one histone acetylation modification was potentiated by CIE in the NAc, CCx, and PFC; additionally, there were no significant differences in histone acetylation in these structures 8–9 h after mice were removed from EtOH vapor. Potentiation of histone acetylation during EtOH exposure and deacetylation during early withdrawal indicates a complex balance between chromatin modifying enzymes that likely depends on blood EtOH levels. Moreover, this pattern implicates early EtOH withdrawal as a critical period for epigenetic remodeling, since the histone modifications studied were altered within 8 h of being removed from EtOH vapor in the NAc and PFC. Interestingly, blood EtOH levels likely remain elevated during this early withdrawal period (Goldstein and Zaechelein, 1983). If EtOH-induced histone modifications diminish while blood EtOH levels are elevated, there may be a role for tolerance in EtOH's epigenetic effects. This role could be studied by correlating blood EtOH levels to induction of histone modifications at multiple time points. A previous study showed increased histone deacetylation 24 h following chronic EtOH exposure in the amygdala (Pandey et al., 2008) and our results indicate these changes occur much earlier in other brain regions. Studying specific windows where large-scale epigenetic changes occur, like the early EtOH withdrawal period identified in this study, will be crucial in finding new epigenetic mechanisms important for EtOH action. Moreover, most studies of EtOH-induced histone deacetylation have used nonspecific HDAC inhibitors like Trichostatin A (TSA) (Pandey et al., 2008; Pascual et al., 2012; Sakharkar et al., 2012; You et al., 2014). Utilizing inhibitors more selective for specific HDACs (Bradner et al., 2010) to modify histone deacetylation during EtOH withdrawal will also help identify these mechanisms.

We identified a significant and transient induction in histone acetylation in NAc and PFC following CIE. Notably, the CIE paradigm escalates EtOH drinking in mice 72 h after EtOH exposure (Becker and Lopez, 2004; Lopez and Becker, 2005; Griffin et al., 2009) and is associated with persistent changes in glutamate signaling in NAc (Griffin et al., 2014). Based on this we predicted EtOH-induced histone modifications would remain altered at this time point. A previous study also reported that 9 days of EtOH vapor resulted in increased acetylation of histone subunits H3 and H4 in the VTA that persisted for 3 days after EtOH exposure (Shibasaki et al., 2011). However, our results indicate that global increases in histone acetylation induced by high BEC's quickly diminish during withdrawal in NAc and PFC. Transient induction of histone acetylation in these structures may be associated with reinforcement of EtOH consumption. Notably, treatment with TSA, which globally increases histone acetylation, leads to escalation of EtOH drinking in mice (Wolstenholme et al., 2011). Induction of histone modifications during but not immediately after EtOH exposure also fits with studies of post-mortem tissue from human alcoholics, which identify only modest changes in gene expression and epigenetic modifications in the brain associated with chronic EtOH use (Zhou et al., 2011; Ponomarev et al., 2012). Along these lines, it is likely that changes in histone acetylation persist at specific gene promoters and mediate effects on gene expression without widespread epigenetic dysregulation. Additional studies of gene expression or ChIP-seq following prolonged withdrawal from EtOH would reveal these effects.

We also identified chromatin modifying enzymes that may mediate induction of histone acetylation in NAc. Increased expression of the histone acetyltransferases (HAT) *Kat2b (Pcaf)* and *Kat5* (*Tip60*) correlates with induction of histone acetylation in NAc following CIE. *Kat2b* was also found to be up-regulated in CCx during an acute EtOH exposure (Finegersh and Homanics, 2014), suggesting it is induced in multiple regions following EtOH exposure. We identified an increase in *Tet1*, which is a dioxygenase involved in active DNA demethylation in the brain (Guo et al., 2011) and suggests CIE contributes to active DNA demethylation in NAc. Notably, 3 weeks of a liquid diet containing 5% EtOH was shown to decrease global DNA methylation in brain tissue (Fowler et al., 2012). Studying the effect of CIE on DNA methylation may reveal a similar effect. We did not identify differential expression of these chromatin modifying enzymes in PFC, which parallels the less significant effect of EtOH on histone acetylation in this region. Still, a previous study reported significantly more differentially expressed genes in PFC compared to NAc following CIE (Melendez et al., 2012), suggesting EtOH-induced induction of chromatin modifying enzymes is important in PFC. Studying expression of additional chromatin modifying enzymes may identify those important for EtOH-induced epigenetic regulation in PFC.

We identified a significant effect of CIE on H3K18ac but not H3K27me3, H3S10pK14ac, H3K9ac, or H3K14ac in CCx, indicating only a modest effect of EtOH on these histone modifications in this brain region. Notably, H3K18ac is found almost exclusively at the transcriptional start site of actively transcribed genes (Wang et al., 2008), so that an increase in this histone modification following chronic EtOH may be associated with globally increased gene expression in CCx. Additionally, lack of a significant effect on other histone modifications in CCx is consistent with our previous study showing acute EtOH exposure did not alter global levels of H3K27me3, H3K9ac, or H3K14ac in CCx but did significantly increase levels of H3K9ac and H3K14ac in the hippocampus (Finegersh and Homanics, 2014). Lack of an effect of EtOH on histone modifications in the CCx in this study may also be due to the use of pyrazole in Experiment 2. Pyrazole inhibits alcohol dehydrogenases and reduces acetaldehyde production. One study found that acetalydehyde potentiated histone phosphorylation in hepatocytes at a faster rate than EtOH alone (Lee and Shukla, 2007), so it is possible acetaldehyde plays a similar role in the brain. Despite the potential effects of pyrazole, our findings suggest that while EtOH induces differential gene expression in CCx (Devaud et al., 1995; Pignataro et al., 2007; Finegersh and Homanics, 2014), this structure likely has greater heterogeneity of epigenetic mechanisms and may be more challenging to study compared to smaller regions like the hippocampus, NAc, or PFC. Subegion-specific differences in expression of neurotransmitter receptors within CCx also supports this point (Jin et al., 2014). Still, given the potential of drugs that target chromatin modifying enzymes for treatment of AUD (Brady et al., 2002; Salloum et al., 2005), investigating epigenetic mechanisms of EtOH in a large and diverse structure like CCx will improve understanding of global epigenetic effects of EtOH and drugs used to modulate EtOH consumption.

Our comparison of α1SHLA and WT mice across multiple EtOH exposures revealed an effect of genotype on levels of H3K27me3, H3K14ac, and H3K18ac as well as a trend for an effect on H3S10pK14ac. Since there were no significant differences in levels of histone modifications between WT and α1SHLA room air controls, this finding suggests EtOH exposure leads to global, subtle changes in histone modifications in CCx of this strain. This finding is consistent with minimal differences in basal activity and altered response to EtOH reported in this strain (Borghese et al., 2006; Werner et al., 2006). It is also supported by altered expression of several GABA_A_ receptor subunits in this strain (Borghese et al., 2006; Harris et al., 2011). Administering HDAC inhibitors or other chromatin modifying enzymes during EtOH exposure would reveal whether epigenetic mechanisms mediate altered sensitivity to EtOH in this strain.

While our study examined the effects of EtOH on six unique histone modifications, over 100 have been identified with most having an unclear function (Tan et al., 2011). Based on the results of this study, histone acetylation during CIE and deacetylation during early withdrawal appear to be key epigenetic mechanisms of EtOH action in the NAc, PFC, and CCx. This finding parallels similar roles of EtOH-induced histone acetylation and withdrawal-induced deacetylation in the amygdala (Pandey et al., 2008; Sakharkar et al., 2012; Moonat et al., 2013). However, the role of EtOH on other histone modifications, including repressive marks like H3K27 and H3K9 methylation is not clear. These repressive histone modifications play a role in silencing retrotransposable and repetitive elements in the genome (Wang et al., 2008), which were recently discovered to be upregulated in post-mortem tissue of human alcoholics (Ponomarev et al., 2012). Therefore, studying histone methylation and other modifications will be critical for identify how EtOH alters chromatin to bring about changes in expression.

In conclusion, we show that histone modifications induced by EtOH are sensitive to changes in genotype, brain region studied, and timing of exposure. These results have significant implications for designing studies of epigenetic mechanisms of EtOH and improve understanding of how EtOH regulates gene expression.

### Conflict of interest statement

The authors declare that the research was conducted in the absence of any commercial or financial relationships that could be construed as a potential conflict of interest.
